# Complication rare de la coloscopie chez un patient sous anticoagulant: hemopéritoine par rupture d'un hématome sous capsulaire de la rate, cas clinique

**DOI:** 10.11604/pamj.2015.21.232.1920

**Published:** 2015-07-31

**Authors:** Hicham Sbai, Brahim Boukatta, Abderahim El Bouazzaoui, Mounia Youssfi, Ihsane Mellouki, Dafr Allah Benajeh, Meriem Bobo, Hicham Bohadouti, Siham Tizniti, Adil Ibrahimi, Khalid Ait Taleb, Nabil Kanjaa

**Affiliations:** 1 Service d'Anesthésie Réanimation A4, CHU Hassan II, Fès, Maroc; 2 Service de Gastroentérologie, CHU Hassan II, Fès, Maroc; 3 Service de Chirurgie Viscérale, CHU Hassan II, Fès, Maroc; 4 Service de Radiologie, CHU Hassan II, Fès, Maroc

**Keywords:** Hématome sous-capsulaire de la rate, coloscopie, anticoagulant, splénectomie, réanimation, subcapsular hematoma of the spleen, colonoscopy, anticoagulant, splenectomy, reanimation

## Abstract

La coloscopie à visée diagnostique et/ou thérapeutique est un examen invasif fréquemment pratiquée de nos jours. La perforation colique et l'hémorragie digestive en sont les principales complications. La survenue d'un hémopéritoine par rupture d'un hématome sous-capsulaire splénique est une complication extrêmement rare et potentiellement mortelle de la coloscopie. Un traumatisme splénique minime passé inaperçu et la prise d'anticoagulant en sont des facteurs favorisants. Nous présentons le cas d'une rupture d'un hématome sous-capsulaire de la rate après une coloscopie, survenue chez un patient de 70 ans porteur d'une valve mitrale mécanique sous acénocoumarol à dose hypocaogulante. La nécessité d'obtention d'une anti coagulation rapidement efficace et l'instabilité hémodynamique avaient justifiée la réalisation d'une splénectomie. L’évolution était favorable. A travers cette observation clinique nous discutons les mécanismes et les modalités de prise en charge devant cette complication.

## Introduction

L'hématome sous-capsulaire de la rate est une complication exceptionnelle de la coloscopie. Le traumatisme de la rate par traction sur l'entrecroisement ligamentaire péri splénique, est le principal mécanisme invoqué [1]. L'altération de l'hémostase secondaire à un traitement anticoagulant, prescrit pour une cardiopathie ou une prothèse valvulaire, est un facteur favorisant le saignement. Le tableau clinique est parfois trompeur car l'instabilité hémodynamique peut faire évoquer un choc cardiogénique en raison de la pathologie cardiaque sous-jacente.

## Patient et observation

Un homme de 70 ans était hospitalisé au service de gastroentérologie pour bilan étiologique d'un syndrome anémique secondaire à un mélaena de faible abondance qui remontait à 3mois sans retentissement hémodynamique. Dans ses antécédents, on trouvait un remplacement valvulaire mitral par une valve mécanique depuis 20 ans sous acénocoumarol (Sintrom^®^) à dose hypocoagulante et une hernie inguinale bilatérale (droite opérée il y a 4 ans et gauche découverte depuis un an). L'examen clinique avait objectivé des traces de mélaena au toucher rectal sans autres sites de saignement et le bilan biologique d'hémostase n’était pas en faveur d'un surdosage à l'antivitamine K (AVK): INR (International Normalized Ratio) à 2,8.

Un scanner abdominal réalisé avant l'endoscopie n'a pas révélé de processus tumoral, d'adénopathies ou d’épanchement péritonéal et la rate était sans anomalies. La coloscopie totale, réalisée dans le cadre du bilan étiologique, était marqué par l'impossibilité du passage de l'endoscope à travers le colon sigmoïde. La décision était de refaire une autre coloscopie après la cure de l'hernie inguinale gauche. Cette dernière était marquée par une dissection laborieuse et la découverte d'une grosse hernie du colon sigmoïde adhérent à la paroi scrotale.

Une coloscopie totale était programmée trois semaines après le geste chirurgical. Un relai de l'AVK par l’énoxaparine à dose curative (60 mg deux fois par jour pour un poids de 60Kg), était débuté 5 jours avant le geste endoscopique et arrêtée la veille. Le bilan biologique, réalisé le jour de l'endoscopie, avait trouvé: INR à 1,52, TCA 38"/35" avec un taux d'hémoglobine à 10,4 g/dl sans thrombopénie ou insuffisance rénale. La coloscopie était faite sous sédation par du propofol en anesthésie intraveineuse à objectif de concentration (AIVOC) et des bolus de sufentanyl. L'exploration endoscopique avait duré 35 min et montré une angiodysplasie du colon droit retenue comme cause du saignement nécessitant une électrocoagulation au Plasma Argon. Aucune difficulté technique n’était constatée par l'endoscopiste notamment au passage de l'angle colique gauche; par ailleurs, il n'y avait pas de masse tumorale ou de polypes.

**Figure 1:**
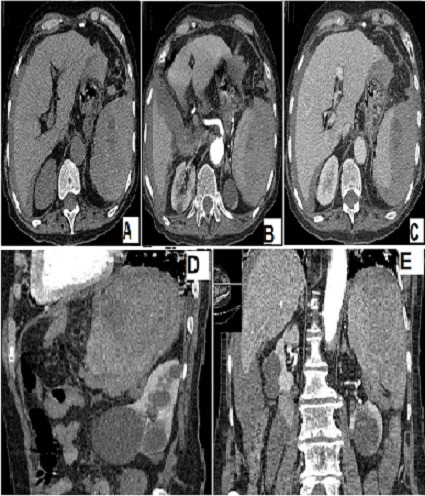
Coupes scannographiques sans injection (A) et avec injection au temps artériel (B) et parenchymateux (C) avec reconstructions sagittale (D) et coronale (E) objectivant un hématome sous-capsulaire splénique spontanément hyperdense associé à un épanchement péri-hépatique

Dix heures après la coloscopie, le patient avait présenté un tableau d'abdomen aigu avec une sensibilité de l'hypochondre gauche et une distension abdominale généralisée associé à des nausées, une pâleur conjonctivale sans syndrome fébrile. Le patient n'avait pas accusé de douleurs thoraciques et l'auscultation cardio-pulmonaire était sans particularité. La tension artérielle était à 100/71mmHg, la fréquence cardiaque à 110 battements/min sans arythmie, la SpO2 à 96% en air ambiant et la fréquence respiratoire à 18 cycles /min. Le traitement anticoagulant n’était pas encore repris après la coloscopie. La prise en charge était assurée au service de réanimation, ou le patient avait reçu une oxygénothérapie au masque faciale, un remplissage vasculaire par de l'hydroxyethyl amidon à 6% après un test de lever de jambe passif positif. L’électrocardiogramme avait objectivé une tachycardie sinusale sans modifications électriques du segment ST. La biologie avait objectivé un taux d'hémoglobine à 8,2g/dl, plaquettes à 185000 éléments / mm^3^, INR à 1,51, TCA à 39"/35", troponine à 0,08, urée à 11,7mmol/l, créatinine à 133µmol/l, lactatémie à 2,7 mmol/l, un bilan hépatique et hydro électrolytique normal et un groupage sanguin A+ avec RAI négatives. Le patient était transfusé par 3 culots globulaires iso groupe iso Rhesus. Les données de l’échocardiographie transthoracique étaient les suivantes: fraction d’éjection à 50%, pas d'anomalies de la valve mécanique mitrale, pas d'hypertension artérielle pulmonaire, pressions de remplissage non élevées, pas de dilatation de l'oreillette gauche ou des cavités ventriculaires et pas d’épanchement péricardique. Le scanner abdominal avait montré un épanchement de moyenne abondance en péri hépatique et au niveau du Douglas sans pneumopéritpoine, un volumineux hématome sous-capsulaire de la rate mesurant 5,5cm d’épaisseur avec une compression sans fracture du parenchyme splénique qui restait vascularisée et une intégrité du hile splénique signant la rupture capsulaire de la rate (Figure 1).

L’évolution était marquée par une déglobulisation du patient (hémoglobine passant à 7,2 g/dl), une altération de l’état hémodynamique avec des signes d'hypovolémie: tachycardie (120 à 130 battement/min), hypotension artérielle (pression artérielle systolique: 80 à 85mmHg), pression veineuse centrale basse (3 - 5 cm H_2O_). Devant l'instabilité hémodynamique et la nécessité de la reprise rapide d'une anticoagulation efficace (étant donné le risque important de thrombose de la valve mécanique), une laparotomie d'hémostase et d'exploration était justifiée après une concertation multidisciplinaire incluant anesthésiste-réanimateur, cardiologue et chirurgien. L'exploration chirurgicale avait montré un hématome sous-capsulaire splénique rompu dans la cavité péritonéale avec un hémopéritoine de 1,3 litres sans autres anomalies. Le geste chirurgical réalisée était une splénectomie d'hémostase avec un lavage péritonéal abondant et un drainage de la loge splénique, des gouttières pariéto-coliques et du Douglas. Des épisodes d'hypotension artérielle étaient observés en per opératoire répondant au remplissage vasculaire et à la transfusion de 3 culots globulaires. Le patient était extubé après réveil complet et réchauffement sans incident cardio-respiratoire. L'analgésie était assurée par une association PCA morphine-proparacetamol. L'antibioprophylaxie assurée par l'amoxicilline-acide clavulanique était maintenue en post opératoire. Le bilan biologique post opératoire avait montré un taux d'hémoglobine à 10,2g/dl, TP à 57%, INR à 1,47, TCA 37"/35", plaquettes 189000 éléments/mm^3^, urée à 8,6mmol/l, créatininémie à 137µmol/l et une troponine à 0,06. Etant donné le contrôle du saignement et la normalité du bilan biologique postopératoire, une anticoagulation par l'héparine sodique à la seringue électrique a été débutée 8heures après la chirurgie. L'hypocoagulation était obtenue par 350 UI/Kg/j avec un TCA à 2,8 fois le témoin sans saignement par les drains ni de déglobulisation ou de thrombopénie. Le Sintrom était débuté à J4 post chirurgie et un INR à 2,9 était obtenu avec un ½ comprimé par jour. Les drains chirurgicaux étaient retirés à j4. L'héparine sodique était arrêtée après 5 jours de chevauchement et après l'obtention de deux INR dans la fenêtre thérapeutique à 24 heures d'intervalle. L'examen histologique de la rate confirmait l'aspect non pathologique. Le patient avait reçu son vaccination antipneumocoque et antihemophilus, et mis sous oracilline orale. La sortie de l'hôpital était autorisée au 11^ème^ jour post opératoire et un suivi régulier en cardiologie était prévu.

## Discussion

La coloscopie, pratiquée à visée diagnostique ou thérapeutique, reste un examen invasif qui n'est pas dépourvu de complications. La perforation colique et l'hémorragie digestive en sont les complications les plus fréquentes. D'autres complications plus rares peuvent s'observer à type de pneumothorax, pneumomédiastin, déchirure mésentérique ou volvulus colique [1, 2]. L'hématome sous-capsulaire de la rate après une coloscopie est une entité exceptionnelle. L'incidence de cette complication est variable dans la littérature et est probablement sous-évaluée: elle va d'un cas pour 6000 coloscopies à aucun cas rapporté sur 30463 examens [3]. Le premier cas rapporté date de 1974 [1, 3]. La prise d'anticoagulants ou de thrombolytiques pour une pathologie cardiaque sous-jacente, est un facteur favorisant certain. Des cas rares de rupture spontanée de la rate ont été rapportés après un traitement anticoagulant ou thrombolytique dont le mécanisme exact n'est pas connu. L'existence d'un traumatisme minime passé inaperçu ou un effort de vomissement survenant dans un contexte d'anti coagulation excessive sont les principaux mécanismes invoqués en l'absence de pathologique splénique sous-jacente [4–6]. Bien que le mécanisme exact du traumatisme splénique lors d'une coloscopie reste inconnu, diverses explications sont avancées. La présence d'adhérences entre le colon gauche, les viscères voisins et la capsule de la rate rend la mobilité du colon gauche amoindrie.

La traction excessive de l'angle colique gauche au moment du passage de l'endoscope provoque un étirement de la capsule splénique par contiguïté avec une traction excessive sur les ligaments gastrocolique et gastrosplénique. Les manœuvres d’étirement du côlon pour faciliter un débouclage avant le franchissement de l'angle colique gauche et la réalisation d'une polypectomie peuvent aussi favoriser la survenue de cette complication. La position en décubitus latéral gauche et les manœuvres de pression sur la paroi abdominale sont également 2 manœuvres suspectées de diminuer la mobilité de la rate et de la rendre plus vulnérable à la traction exercée par l'intermédiaire de l'endoscope [2, 4, 7–8]. D'autres facteurs favorisants ont été rapportés tel qu'une chirurgie de l'hypochondre gauche, une poussée de pancréatite et une maladie inflammatoire de l'intestin [7, 9]. L'amélioration du matériel et, notamment, la souplesse des endoscopes ne semble pas avoir éliminé ce genre de complication. Le traumatisme splénique compliquant une coloscopie peut prendre plusieurs formes: fissure ou rupture du parenchyme, hématome sous-capsulaire sans lésion parenchymateuse, hémopéritoine par déchirure de la capsule splénique.

L'instabilité hémodynamique par hémorragie splénique peut initialement être interprétée comme un choc cardiogénique par infarctus de myocarde chez des patients porteurs d'une cardiopathie avérée. Le tableau clinique abdominal et les données de l’échographie et scanner abdominale redressent le diagnostic. Le délai de diagnostic de cette complication varie de quelques heures à quelques jours après l'endoscopie. Dans une revue de La littérature s'intéressant à 27 des cas décrits, le diagnostic était fait dans les 24premières heures dans 67% des cas [8, 10], ce qui est le cas dans notre observation. La douleur de l'hypochondre gauche irradiant dans l’épaule homolatérale est le plus souvent le signe révélateur, mais ce dernier est peu spécifique car présent chez plus de 50% des patients après une coloscopie non compliquée [11].

Dans notre cas, l'imputabilité de la coloscopie dans la survenue du traumatisme splénique est fortement probable du fait de: la relation chronologique entre la symptomatologie abdominale et le geste endoscopique, l'absence de tout traumatisme thoraco-abdominal externe, l'absence d'anomalie splénique sur le scanner abdominal réalisé avant la coloscopie et la difficulté technique rencontrée lors de la cure récente de l'hernie inguinale gauche avec la découverte de l'hernie du colon sigmoïde. Cette complication était favorisée, chez notre patient, par l'altération de l'hémostase liée à la prise d'anticoagulant sans signes biologiques de surdosage. La prise en charge thérapeutique de cette complication est multidisciplinaire (anesthésiste-réanimateur, radiologue, chirurgien et cardiologue dans notre cas) et doit se faire en milieu de réanimation. La décision d'abstention chirurgicale repose essentiellement sur les critères suivants: la stabilité hémodynamique, l'existence d'un hématome sous-capsulaire sans hémopéritoine ni extravasation de produit de contraste à l'examen tomodensitométrique, l'absence d'autre lésion intra-abdominale imposant une laparotomie et l'absence d'une déglobulisation progressive nécessitant une transfusion sanguine de plus de 2 culots globulaires sous réserve d'une surveillance clinique, biologique et radiologique stricte [9–10].

Les modalités de surveillance par imagerie et le temps d'observation nécessaire en hospitalisation ne sont pas codifiées; le risque étant d’éviter les ruptures tardives après la sortie [12]. Certaines études suggèrent que le traitement conservateur est voué à l’échec si la transfusion de plus d'un culot globulaire est nécessaire [13]. Le taux d’échec du traitement conservateur est de l'ordre de10% et les transfusions sanguines sont plus importantes en cas de traitement conservateur qu'en cas de splénectomie [13]. L'héparinothérapie intraveineuse a ici sa place pour une gestion souple de l'anticoagulation. Elle permet d'assurer une hypo coagulation efficace et rapidement réversible en visant un temps de céphaline activé entre 2,5 et 3 fois le témoin. Devant une instabilité hémodynamique et un surdosage en AVK, la prise en charge doit être rapide et consiste en une antagonisation de l'anticoagulant et une splénectomie. La gestion thérapeutique du surdosage dépend de l'urgence de l'intervention chirurgicale. La perfusion du Kaskadil^®^ (facteurs II,VII,IX,X), à raison de 20 à 30µg/kg exprimées en UI de facteur IX, est recommandée si on a besoin d'une correction immédiate du déficit en facteurs vitamine K dépendants. La vitamine K administrée simultanément par voie orale ou parentérale (10à20mg), permet d'atteindre un niveau de sécurité qui se situe dessous de 1,5 d'INR en six heures, au moment où l'effet du Kaskadil^®^ dont la durée d'action est courte, commence à s’épuiser [4, 6, 14]. La nécessité d'un apport liquidien important (choc hémorragique) et l'absence de disponibilité du concentré complexe prothrombinique, justifient l'utilisation de plasma frais congelés (PFC). La correction du surdosage en AVK par les PFC nécessite de grands volumes et expose donc au risque de surcharge liquidienne pulmonaire chez les porteurs de prothèse valvulaire ou d'autres cardiopathies. Le recours à l'embolisation de l'artère splénique avec pose de coils trouve son indication dans une situation intermédiaire: stabilité hémodynamique, déglobulisation progressive et une extravasation du produit de contraste en tomodensitométrie [11, 15–16].

Dans notre cas, la décision opératoire était basée sur les éléments suivants: l'instabilité hémodynamique, la déglobulisation après transfusion sanguine et la nécessité d'obtenir une anti coagulation rapidement efficace chez un patient à haut risque de thrombose de sa valve mécanique mitrale.

## Conclusion

L'hématome sous-capsulaire de la rate est une complication rare et méconnue de la coloscopie que tout endoscopiste doit connaitre, en raison du nombre croissant de coloscopies réalisées et du caractère potentiellement mortel de cette complication. La survenue retardée d'une douleur de l'hypochondre gauche dans un contexte d'instabilité hémodynamique, ou de déglobulisation dans les heures qui suivent une coloscopie doit y faire penser et amener à réaliser une imagerie abdominale, qui permettra, en outre, d’écarter le diagnostic de perforation colique. La prise d'anticoagulant doit faire rapidement penser au diagnostic. La prise en charge optimale nécessite une concertation multidisciplinaire et doit être assurée dans un service de soins intensif que ce soit pour une surveillance ou une éventuelle sanction chirurgicale.
